# Comparison of targeted next-generation sequencing and Sanger sequencing for the detection of *PIK3CA* mutations in breast cancer

**DOI:** 10.1186/s12907-015-0020-6

**Published:** 2015-11-18

**Authors:** Ruza Arsenic, Denise Treue, Annika Lehmann, Michael Hummel, Manfred Dietel, Carsten Denkert, Jan Budczies

**Affiliations:** Institute of Pathology, Charité University Hospital Berlin, Berlin, Germany

**Keywords:** Next-generation sequencing, Breast cancer, Sanger sequencing, *PIK3CA*

## Abstract

**Background:**

Phosphatidylinositol-4,5-bisphosphate 3-kinase, catalytic subunit alpha, *PIK3CA,* is one of the most frequently mutated genes in breast cancer, and the mutation status of *PIK3CA* has clinical relevance related to response to therapy.

The aim of our study was to investigate the mutation status of PIK3CA gene and to evaluate the concordance between NGS and SGS for the most important hotspot regions in exon 9 and 20, to investigate additional hotspots outside of these exons using NGS, and to correlate the *PIK3CA* mutation status with the clinicopathological characteristics of the cohort.

**Methods:**

In the current study, next-generation sequencing (NGS) and Sanger Sequencing (SGS) was used for the mutational analysis of *PIK3CA* in 186 breast carcinomas.

**Results:**

Altogether, 64 tumors had *PIK3CA* mutations, 55 of these mutations occurred in exons 9 and 20. Out of these 55 mutations, 52 could also be detected by Sanger sequencing resulting in a concordance of 98.4 % between the two sequencing methods. The three mutations missed by SGS had low variant frequencies below 10 %. Additionally, 4.8 % of the tumors had mutations in exons 1, 4, 7, and 13 of *PIK3CA* that were not detected by SGS. *PIK3CA* mutation status was significantly associated with hormone receptor-positivity, HER2-negativity, tumor grade, and lymph node involvement. However, there was no statistically significant association between the *PIK3CA* mutation status and overall survival.

**Conclusions:**

Based on our study, NGS is recommended as follows: 1) for correctly assessing the mutation status of *PIK3CA* in breast cancer, especially for cases with low tumor content, 2) for the detection of subclonal mutations, and 3) for simultaneous mutation detection in multiple exons.

**Electronic supplementary material:**

The online version of this article (doi:10.1186/s12907-015-0020-6) contains supplementary material, which is available to authorized users.

## Background

Historically, Sanger sequencing (SGS) has been the gold standard for detecting DNA mutations. However, SGS has limitations due to its restricted sensitivity and its inability to perform parallel investigation of multiple targets. Furthermore, somatic cancer mutations can be difficult to detect using SGS without performing microdissections because tumors are heterogeneous and often mixed with normal tissue. Recent progress in massive parallel sequencing, termed next-generation sequencing (NGS), has increased the speed and efficiency of mutation testing in molecular pathology [1–9]. NGS allows for the detection of a broad spectrum of mutations, including single nucleotide substitutions, small insertions and deletions, large genomic duplications and deletions, and rare variations [9].

Targeted NGS, which involves the targeted enrichment of a set of DNA regions, is used for the parallel sequencing of amplicons derived from multiplex polymerase chain reaction (PCR) or other amplicon-based enrichment approaches, such as hybridization capture. When the amplicon size is kept small (e.g., <175 bp) in the design of the sequencing panel, NGS is also applicable to formalin-fixed tissue samples. Moreover, targeted NGS is more cost efficient than SGS [10]. This high-throughput technology is currently used with several platforms, including the Genome Analyzer/HiSeq/MiSeq (Illumina Solexa), the SOLiD System (Thermo Fisher Scientific), the Ion PGM/Ion Proton (Thermo Fisher Scientific), and the HeliScope Sequencer (Helicos BioSciences) [11, 12].

NGS can be used to detect both somatic and germline mutations in the cancer genome. The somatic genetic changes can be classified as either driver or passenger mutations. The former contribute to tumor development [13, 14], while the latter do not directly contribute to tumor development and may be the product of genomic instability within the tumor. Although SGS and PCR are routinely used to identify clinically relevant mutations and select the best treatment for patients, these techniques are insensitive to changes occurring at an allele frequency lower than 20 %, apart from real-time PCR, which could reach higher sensitivity [15, 16].

However, the more sensitive and cost-effective multiplex NGS testing platforms provide comprehensive genomic information, and thus allow for the implementation of targeted therapies and improved treatment decisions [17]. *TP53* and *PIK3CA* are the most frequently mutated genes in breast cancer (BC), both being mutated in about one-third of all primary breast carcinomas [18, 19]. In recent years, several studies identified the clinical relevance of *PIK3CA* mutations in terms of decreasing the benefits of anti-HER2 therapies and poly-chemotherapies in patients with *PIK3CA* mutations [20–22] . In the present study, we investigated the *PIK3CA* status of 186 primary BC patients from the Berlin area using targeted NGS and SGS. Recent studies have analyzed mutations in hot spots (i.e., exon 9 and 20) and only a few studies have analyzed mutations in other exons [23] Consequently, our aims were to evaluate the concordance between NGS and SGS for the most important hotspot regions in exon 9 and 20, to investigate additional hotspots outside of these exons using NGS, and to correlate the *PIK3CA* mutation status with the clinicopathological characteristics of the cohort.

## Methods

### Patient cohort and histopathological evaluation

Tissue samples were collected from 186 patients with a diagnosis of primary BC at the Department of Pathology, University Hospital Charité and the Breast Cancer Center at the DRK Klinikum Koepenick in Berlin, Germany. The median follow-up time was 38 months. Data on tumor histology and tumor grade were evaluated at the time of primary diagnosis and extracted from pathology reports. Tumors were graded according to the Bloom-Richardson grading system modified by Elston and Ellis [24]. HER2 status was determined by immunohistochemistry (IHC) using the Dako HercepTest kit (Dako, Carpinteria, CA, USA). Chromatic in situ hybridization (CISH) was also performed on samples with a HER2 score of 2+. The estrogen receptor (ER) monoclonal antibody clone SP1 (NeoMarkers, Fremont, CA, USA) was used to identify the ER status, and the progesterone receptor (PR) status was determined with the PR monoclonal antibody PgR 636 (Dako, Wiesentheid, Germany). Only nuclear labeling was scored as positive. Negative ER and PR status was defined as positivity in <1 % of tumor cells according to ASCO/CAP guidelines [25]. HER2 negativity (HER2-) was defined as the absence of membranous staining or weak, discontinuous membranous staining. Cases with moderate membranous staining in >10 % of the tumor cells were examined by CISH according to ASCO/CAP guidelines [26]. A proliferation index was not available for all samples. Representative tumor samples containing at least 30 % tumor cells were selected for molecular studies.

### Sample cohort and clinical parameters

Median patient age at the time of diagnosis was 65 years, with a range of 34–95 years.

A total of 149 patients (80.1 %) had ductal carcinoma and 20 (10.7 %) had lobular carcinoma. Seventeen patients (9.1 %) had carcinoma of another histological type, such as mucinous ductal carcinoma with squamous differentiation, mixed-ductal and lobular carcinoma, medullary carcinoma, or invasive papillary adenocarcinoma. None of the patients received any medical treatment related to BC before surgery. After diagnosis, most (93 %) of the hormone receptor-positive (HR+) cases were administered hormonal therapy alone or in combination with other therapies according to relevant guidelines.

## Ethics approval

Patients provided written informed consent for use of their biomaterial samples in biomarker studies. Consent was obtained using the standardized informed consent forms of the participating institutions. The project and consent process was approved by the ethics board of the Charité Hospital, Berlin (reference number EA1/139/05, last amendment 2013).

### DNA extraction, PCR, and *PIK3CA* semiconductor next-generation sequencing

Briefly, 10 consecutive 10-μm thick sections were prepared. The first section was stained with hematoxylin/eosin and the tumor area was marked by a pathologist. The corresponding area was manually microdissected from each of the consecutive unstained sections and transferred to 180 μl of lysis buffer (QIAamp® DNA Mini Kit, Qiagen, Venlo, Niederlande) for 10 min at 95 °C. Enzymatic lysis was carried out with 20 μl of Proteinase K for 1 h at 56 °C. Subsequent DNA preparation was performed according to the manufacturer’s instructions, and the DNA was eluted in 80 μl of elution buffer. Total nucleic acid concentrations were measured with a Qubit fluorometer HS DNA Assay (Life Technologies, Carlsbad, CA, USA) and a TaqMan RNase P Detection Reagents Kit (Life Technologies). Ten nanograms of genomic DNA were utilized for the library preparation. The final library was quantified using an Ion AmpliSeq Library Kit 2.0 (Life Technologies). The samples were 8-fold multiplexed and amplified on Ion Spheres Particles using the Ion OneTouch™ 200 Template Kit v2 DL (Life Technologies). After library enrichment and quality control on a Qubit instrument (Ion Sphere Quality Control Kit, Life Technologies), the samples were sequenced using the Ion 318 chip v2 according to the standard protocol of the chip manufacturer.

A customized sequencing panel consisting of 154 amplicons from 48 genes was designed using the Ion AmpliSeq Designer to cover the most frequent somatic mutations found in BC. The panel included six amplicons located in *PIK3CA* exons 1, 4, 7, 9, 13, and 20. The genomic positions and primer sequences can be found in the well plate data sheet generated by the Ion AmpliSeq Designer (Additional file 1: Table S1). Only samples with at least 30 % tumor cells within the dissected area were included in the study.

Base calling and alignment to the human genome (hg19) were executed with the Torrent Suite Software 4.0.3. The mean coverages (minimum – maximum) of the amplicons were 4128 bp (1315–22668 bp) for exon 1, 5237 bp (1962–25371 bp) for exon 4, 2044 bp (347–12066 bp) for exon 7, 3588 bp (278–20808 bp) for exon 9, 3742 bp (1386–20063 bp) for exon 13, and 1552 bp (438–9901 bp) for exon 20. Variant calling was executed using the Torrent Variant Caller 4.2 and the low stringency somatic variant calling protocol. Only non-synonymous nucleotide exchanges were considered for the analysis of single nucleotide polymorphisms.

### Sanger sequencing primers and sequencing parameters

Primers were designed using the Primer Design Tool from NCBI. The primers were as follows:

- exon 9 forward 5´-GGGAAAAATATGACAAAGAAAGC-3´,
- exon 9 reverse 5´-GAGATCAGCCAAATTCAGTT-3´,
- exon 20 part 1 forward 5´-CATTTGCTCCAAACTGACCA-3´,
- exon 20 part 1 reverse 5´-TgTgCATCATTCATTTgTTTCA-3´,
- exon 20 part 2 forward 5´-TTGATGACATTGCATACATTCG-3´,
- and exon 20 part 2 reverse 5´-GGTCTTTGCCTGCTGAGAGT-3´.

The sequencing reactions were loaded on the 3730xl DNA Analyzer from Hitachi (Applied Biosystems). Sequence traces from tumor DNA samples were aligned to the genomic reference sequence and analyzed using SeqPilot software (Applied Biosystems).

### Statistical evaluation

Statistical analyses were conducted using the SPSS 19 statistical software (SPSS Inc., Chicago, IL, USA) and the statistical language R (Foundation for Statistical Computing, Vienna, Austria). Significance of associations between *PIK3CA* status and age, ER/PR status, tumor stage, and histological grade were assessed using a Fisher’s exact test, a chi-squared test, and a chi-squared test for trends. Overall survival was analyzed using the Kaplan-Meier method and the log-rank test. All tests were two-tailed, and results were considered significant when *p* < 0.05. Barplots and beeswarm plots were produced using the R package graphics and beeswarm [27, 28].

## Results

### Prevalence of different types of *PIK3CA* mutations using NGS

**Fig. 1:**
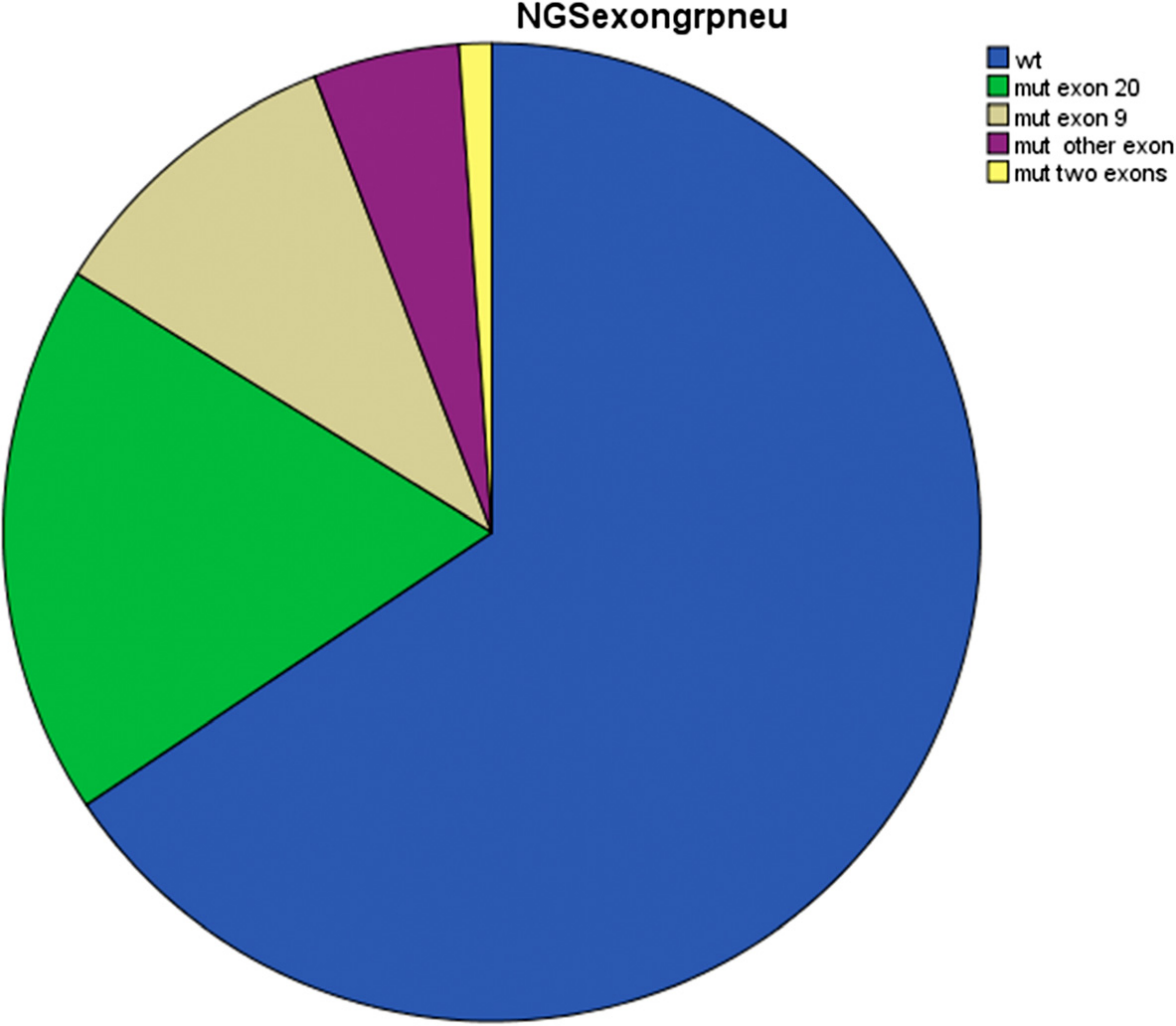
*PIK3CA* mutations in breast cancer: frequency of mutations, including information on the affected exons

Using NGS to sequence the *PIK3CA* gene in each of the 186 tissue samples identified a total of 64 tumors with exon mutations (34.4 %), which agreed with the 36 % *PIK3CA* mutation rate in BC reported by the Cancer Genome Atlas (TCGA) [18, 19]. As shown in Fig. 1, the mutations were distributed as follows: exon 20 (34 cases; 18.3 %), exon 9 (19 cases; 10.2 %), and other exons (1, 4, 7, or 13) (9 cases; 4.8 %). In very few samples (2 cases; 1.1 %), we found mutations in two exons. The majority of mutations were base pair substitutions (60 cases, 93.8 %). Additionally, we detected deletions (4 cases, 6.3 %). Sixty of the tumors (93.7 %) had a single mutation in the *PIK3CA* gene, while three tumors had two mutations in the *PIK3CA* gene. The most frequent mutations were p.H1047R (31 cases, 48.4 %), p.E545K (11 cases, 17.2 %), and p.E542K (6 cases, 9.4 %) (Table 1).

**Table 1:** Hotspots of *PIK3CA* mutations in breast cancer

Exon	Mutation	Number	Frequency (%)	Total number
1	R108del, R109del	1, 2	1.6, 3.1	3
4	N345K, N347T (new), D350N	3,1,1	4.7, 1.6, 1.6	5
7	G451_D454del (new), p.L456V (new)	1,1	1.6, 1.6	2
9	E542K, E545K, Q546K, Q546R	6, 11, 1, 1	9.4, 17.2	19
13	E726K	2	3,1	2
20	D1029H, N1044K, H1047L, H1047R, G1049R	1, 1, 3, 31, 1	1.6, 1.6, 48.4, 4.7, 1.6	37
		total: 68		

Using semiconductor NGS, we identified a total of 68 non-silent mutations in 186 breast cancer samples (34.4 %). The most frequent aberrations were p.H1047R, p.E545K, and p.E542K

**Fig. 2:**
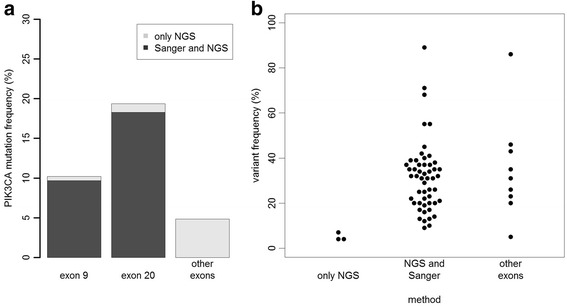
Detection sensitivity of semiconductor NGS compared to Sanger sequencing. **a** Barplot showing the number of mutations detected by both Sanger sequencing and NGS and the mutations detected only by NGS. **b** Beeswarm plot showing the variant frequencies of the mutations detected only by NGS and those detected by both methods

Three mutations, p.N347T, p.G451_D454del, and p.L456V, were not described in any of the studies reported in COSMIC (http://cancer.sanger.ac.uk/cancergenome/projects/cosmic), TCGA [18, 19], or any other large genomic database [29, 30]. Thus, they are being described for the first time in our study. Additionally, a single nucleotide polymorphism (rs3729674) (NC_000003.11:g.178917005A > G) was found in 33 cases. Out of a total of 55 mutations in exons 9 and 20 that were detected using NGS, 49 were also detected by SGS. By resequencing discrepant cases using SGS, we found an additional three cases with mutations with small peak heights that could be detected by analyzing the electropherograms manually. A comparison of the NGS and SGS results is shown in Fig. 2. The three mutations in exon 9 and exon 20 that were missed by SGS had low variant frequencies of 4 % (twice) and 7 %.

### *PIK3CA* mutations and clinical characteristics

**Fig. 3:**
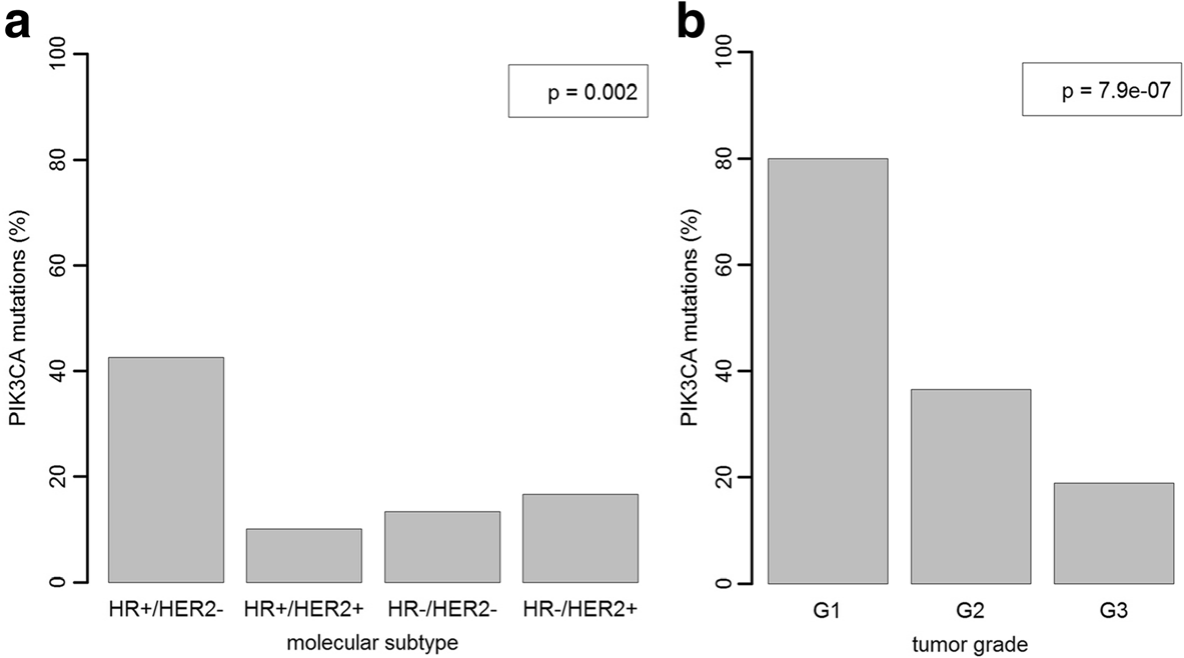
Strong association of *PIK3CA* status with molecular subtype and tumor grade in breast cancer. **a** *PIK3CA* mutations were more frequent in HR+/HER2- breast cancer (40 %) compared with the other subtypes (11 %-20 %). **b** The mutation frequency decreased with increasing tumor grade: 80 % in G1, 36 % in G2, and 19 % in G3

*PIK3CA* mutations were analyzed for correlation with several clinicopathological parameters at the time of the diagnosis: age, tumor size, tumor grade, nodal status, HR status, HER2 expression, and histological subtype (Table 2). *PIK3CA* mutations were most frequently found in HR+, HER2- (*p* = 0.002), and well-differentiated (G1; *p* < 0.001) cases (Fig. 3a and b). Furthermore, there was a statistically significant difference between cases with different nodal statuses; there were more cases with *PIK3CA* mutations in the N1 group (*p* = 0.042). We found no statistically significant correlation between mutation status and age, tumor size, or histological tumor type.

**Table 2:** Correlation of *PIK3CA* mutation status with the clinicopathological characteristics of breast cancer

Clinicopathological parameters	Mutated (%)	Wild type (%)	*P*
All Tumor Cases	64 (34.4)	122 (65.6)	NS
Histological Type			NS
Ductal/Other Carcinoma	53 (32.5)	110 (67.5)	
Lobular Carcinoma	11 (47.8)	12 (52.2)	
Tumor Stage			NS
T1	13 (30.2)	28 (65.1)	
T2	37 (34.9)	69 (65.1)	
T3	9 (42.9)	12 (57.1)	
T4	5 (41.7)	7 (58.3)	
Node Status			0.042
N0	25 (27.8)	65 (72.2)	
N+	37 (42.5)	50 (57.5)	
Tumor Grade			<0.001
G1	16 (80.0)	4 (20.0)	
G2	35 (36.5)	61 (63.5)	
G3	13 (18.8)	56 (81.2)	
Hormone Receptor Status			0.002
HR+	58 (40.3)	86 (59.7)	
HR-	6 (14.3)	36 (85.7)	
HER2 Status			0.032
HER2+	3 (13.6)	19 (86.4)	
HER2-	61 (37.2)	103 (62.8)	
Age			NS
<50 years	6 (26.1)	17 (73.9)	
>50 years	58 (35.6)	105 (64.4)	
Molecular Type			0.003
HR+/HER2-	57 (42.5)	77 (57.5)	
HR+/HER2+	1 (10.0)	9 (90.0)	
HR-/HER2+	2 (16.7)	10 (83.3)	
HR-/HER2-	4 (13.3)	26 (86.7)	

The mutation frequency, as determined by NGS, decreased with increasing tumor grade: 85 % for G1, 37 % for G2, and 20 % for G3. *PIK3CA* mutations were more frequently detected (42 %) in HR+ breast cancer than in HR- breast cancer (14 %). *PIK3CA* mutations were more frequently detected in HER2- breast cancer (38 %) than in HER2+ breast cancer (14 %)

When we compared mutations in the other exons (1, 4, 7, and 13) with the clinicopathological parameters, we found a trend toward enrichment of the mutations in lower grade tumors, but this trend did not reach statistical significance (*p* = 0.104). There was no statistically significant difference between *PIK3CA* mutation status in these other exons and cases that were HR+, HR-, HER2+, or HER2-. Also, we could not detect differences in nodal status, tumor size, or age.

**Fig. 4:**
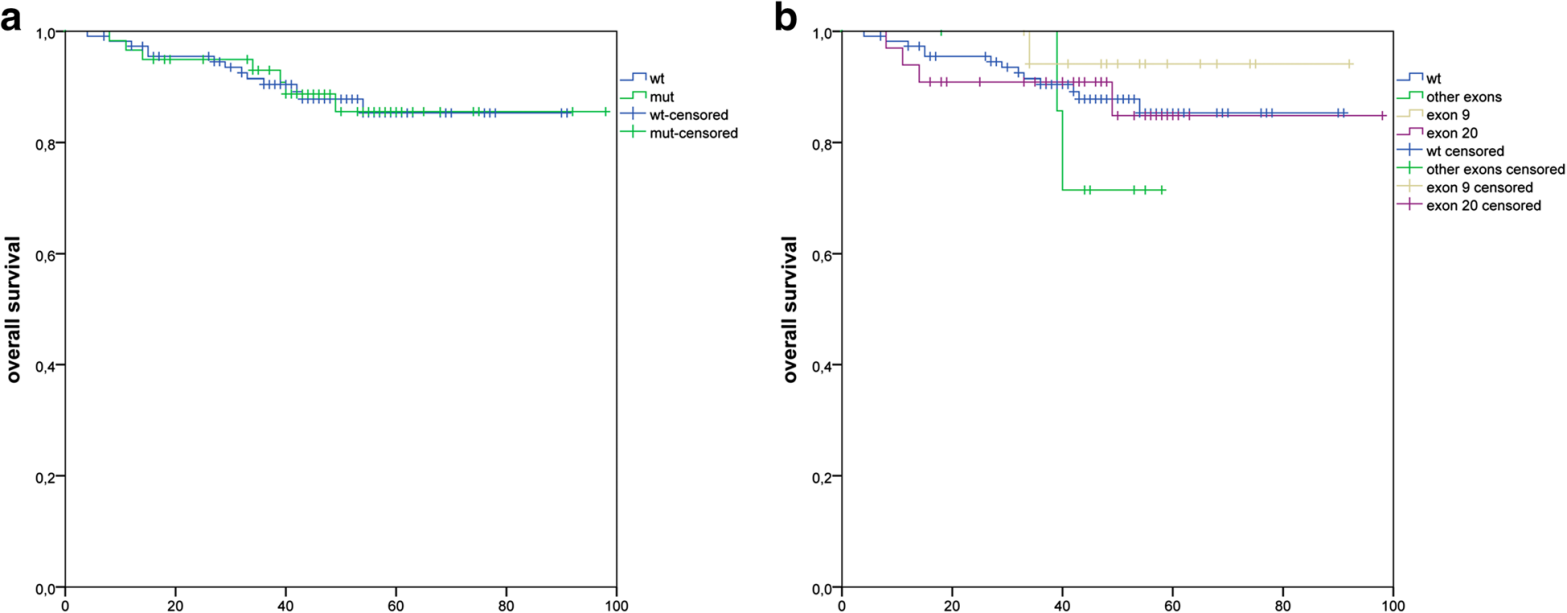
Correlation of overall survival with the *PIK3CA* mutation status in breast cancer. **a** Kaplan-Meier analysis comparing patients with mutated *PIK3CA* (green line) and wild type *PIK3CA* (blue line) did not reveal a statistically significant difference in survival. **b** Kaplan-Meier analysis of the *PIK3CA* mutation status stratified for the affected exon did not reveal a statistically significant difference in survival, but there was a trend toward better survival for the cases with a mutation in exon 9. Exon 9 mutation (*p* = 0.39) vs. exon 20 mutation (*p* = 0.41) vs. other exon mutations (*p* = 0.16)

An overall survival analysis was performed on 184 patients with available follow-up data. In this group, no statistically significant association was found between long-term survival and the *PIK3CA* mutation status (Fig. 4a). There was also no difference in overall survival related to the mutational status of different *PIK3CA* exons (Fig. 4b).

## Discussion

The precise identification of genomic alterations is crucial for personalized cancer therapy. Molecular testing for mutations in cancer susceptibility genes is mainly performed using SGS of individual exons after PCR. The detection threshold of SGS requires an allele frequency of approximately 20 % [31], but BC tissue is heterogeneous, consisting of tumor, stromal, and inflammatory cells, leading to a varying proportion of tumor cells ranging from 20-95 % [32]. Therefore, we speculated that a significant proportion of *PIK3CA* mutations are missed by SGS. There are studies available from other organ systems, including the lung, which report a higher detection rate of mutations using NGS rather than SGS [33, 34]. In general, the detection sensitivity of NGS reported in previous studies ranges from 94–99.9 % [35–39], which is above the sensitivity of Sanger Sequencing. Accordingly, the aim of the present study was to use NGS for the analysis of *PIK3CA* mutations to determine whether additional changes could be identified that might lead to better correlation of the clinicopathological characteristics of breast tumors with *PIK3CA* mutations. We also sought to evaluate the prognostic significance of *PIK3CA* mutation status as previously reported by several authors [40–44]. To this end, we compared the performance of NGS and SGS in the same cohort of patients, and identified *PIK3CA* mutations in 34.4 % of breast tumors using NGS, which is in agreement with the mutation rate reported in TCGA [45].

Overall, we were able to report a good degree of concordance between the two sequencing methods. Only three mutations that were detected by NGS could not be found by SGS due to low variant frequencies below 10 %. Nevertheless, this finding indicates that NGS is more sensitive than SGS, particularly for the detection of low frequency mutations. This has also been reported in previous studies. Rohlin et al. [46] showed that Sanger-based sequencing techniques have problems picking out “minority” gene sequences (mutations below 15 %). Meanwhile, Walsch et al. performed a study on 300 high-risk BC families that screened for mutations in hotspots, and found previously undetected changes in 52 probands [47]. These results clearly support the use of targeted sequencing because it is more sensitive than SGS when it comes to identifying low frequency mutations. Our study, which is the first to compare NGS and SGS for sequencing the *PIK3CA* gene in BC, adds support for this viewpoint. Additionally, the results of our study showed that ~5 % of the mutations were in other exons, and the best and most cost-effective method for detecting these mutations was to use parallel sequencing or targeted NGS. Due to the low mutation rate in these other exons (1, 4, 7, and 13) and a lack of statistical significance when correlated with clinicopathological data, we abstained from validating these mutations by SGS. The clinical relevance of *PIK3CA* mutations outside exon 9 and 20 should be further investigated in future studies.

The *PIK3CA* mutations detected by NGS in our study clustered in two previously reported “hotspot” regions in exons 9 and 20, with most of the mutations clustering in exon 20, which is in agreement with the SGS results reported in our previous study [48]. The consequences of each mutation on the function and regulation of *PIK3CA* requires further consideration. The three novel mutation detected in our study are located in the C2 domain of the PIK3CA gene. The C2 is often involved in phospholipid membrane binding, consequently it is possible that these mutations lead to increased membrane binding, as extensively discussed in the study by Ikenoue at al. [49]. In the study by Gymnopoulos at al, the authors showed that the mutants in C2 domain increase basic positive surface charge of that domain and may therefore mediate improved recruitment of p110α to the cell membrane, making lipid kinase activity independent of signals transmitted through the regulatory subunit, p85 [50]. One of these three mutations, p.L456V is predicted to be probably damaging with a score of 0.988 (sensitivity: 0.27; specificity: 0.99) when analyzed with polyPhen-2 prediction programe.

The goal of our previous study was to analyse only exon 9 and 20 mutations, hence we could not detect these three mutations in this study.

We also noted the presence of multiple mutations in four cases, which has not been reported previously. The significance of these double mutants is unknown, but it is possible that these tumors are multiclonal, and a second hit was required to provide a selective growth advantage if the first mutation was a less potent activator of the kinase.

We found that *PIK3CA* was most frequently mutated in cases that were HR+ and HER2-, which agrees with previously published data [51, 52]. We found significantly more *PIK3CA* mutations in G1 tumors suggesting that theses mutations occur early in BC development, which has also been shown in other studies [53]. Additionally, *PIK3CA* mutations were highly correlated with lymph node status (N+), which is one of the clinical markers associated with patient survival and response to therapy [54, 55]. The finding that *PIK3CA* mutations are more commonly found in HR+ tumors may point to differences in pathogenesis and disease progression between HR+ and HR- tumors. Furthermore, the correlation between *PIK3CA* mutations and lymph node metastasis suggests that activation of the PI3K/Akt pathway may increase the invasion of cancer cells into the lymph nodes. This is supported by the fact that PIP3 regulates cell mobility [56].

There is controversy regarding the prognostic significance of *PIK3CA* mutations. Cizkova et al. [57] described more favorable metastasis-free survival in patients with *PIK3CA* mutations, but Jensen et al. [22] and Baselga et al. [58] reported reduced survival rates and worse outcomes. The largest published study evaluated the *PIK3CA* genotype in 687 tumor samples from patients enrolled in a prospective phase III clinical trial. Those with *PIK3CA* mutations had a better prognosis for the first three years compared to those carrying wild type *PIK3CA* alleles, but this difference disappeared with a longer follow-up [59,60]. In our study, there was no significant association between *PIK3CA* mutational status and overall survival, indicating that an activated *PIK3CA* pathway alone is not a prognostic factor for BC.

An interesting finding by Fu at al. showed that *PIK3CA*-activating mutations are associated with better outcomes in ER+ patients receiving endocrine therapy [61]. This agrees with the observation that *PIK3CA* mutations are more frequent in luminal A tumors compared with luminal B tumors (e.g., 45 % vs. 29 % in the TCGA cohort) [45]. In contrast, in HER2+ BC, several reports show that *PIK3CA* mutations predict adverse outcomes after treatment with trastuzumab [20, 21]. As such, the impact of *PIK3CA* mutations on the clinical outcome of BC seems to vary with the background of other genomic alterations such as HER2 status.

*PIK3CA* mutations also appear to have a significant interest in the prediction of response to targeted therapies, as many drugs specifically targeting PI3K or other effectors of the PI3K/AKT pathway are intended to be administered only to patients with tumor bearing a mutation of *PIK3CA*, which makes the somatic mutations detection more and more important [62].

In summary, our results show that NGS is more sensitive than SGS for detecting *PIK3CA* mutations in BC samples, and that *PIK3CA* mutations are significantly related to HR and HER2 expression status and tumor grade. Further studies are needed to systematically explore the functional relevance of *PIK3CA* mutations and the contribution of PIK3CA mediated activation of the downstream and upstream signaling pathways in breast tumor development and progression.

## Conclusions

1. This is the first paper in which NGS and SGS were compared sequencing PIK3CA gene in breast cancer.
2. We found overall a good concordance between the two methods (98,4 %), but better sensitivity of NGS when it comes to identifying low frequency mutations(<10 %).
3. *PIK3CA* mutation status in breast cancer correlated strongly with HR+ and HER2-, and N1 + .

## Additional file

Additional file 1: Table S1. Genomic positions and the primer sequences. (XLS 45 kb)
